# The Skull’s Girder: A Brief Review of the Cranial Base

**DOI:** 10.3390/jdb9010003

**Published:** 2021-01-23

**Authors:** Shankar Rengasamy Venugopalan, Eric Van Otterloo

**Affiliations:** 1 Iowa Institute for Oral Health Research, College of Dentistry, University of Iowa, Iowa City, IA 52242, USA; shankar-venugopalan@uiowa.edu; 2 Department of Orthodontics, College of Dentistry, University of Iowa, Iowa City, IA 52242, USA; 3 Department of Anatomy and Cell Biology, Carver College of Medicine, University of Iowa, Iowa City, IA 52242, USA; 4 Department of Periodontics, College of Dentistry, University of Iowa, Iowa City, IA 52242, USA

**Keywords:** neural crest, mesoderm, cranial base, craniofacial, endochondral

## Abstract

The cranial base is a multifunctional bony platform within the core of the cranium, spanning rostral to caudal ends. This structure provides support for the brain and skull vault above, serves as a link between the head and the vertebral column below, and seamlessly integrates with the facial skeleton at its rostral end. Unique from the majority of the cranial skeleton, the cranial base develops from a cartilage intermediate—the chondrocranium—through the process of endochondral ossification. Owing to the intimate association of the cranial base with nearly all aspects of the head, congenital birth defects impacting these structures often coincide with anomalies of the cranial base. Despite this critical importance, studies investigating the genetic control of cranial base development and associated disorders lags in comparison to other craniofacial structures. Here, we highlight and review developmental and genetic aspects of the cranial base, including its transition from cartilage to bone, dual embryological origins, and vignettes of transcription factors controlling its formation.

## 1. Introduction

**Figure 1:**
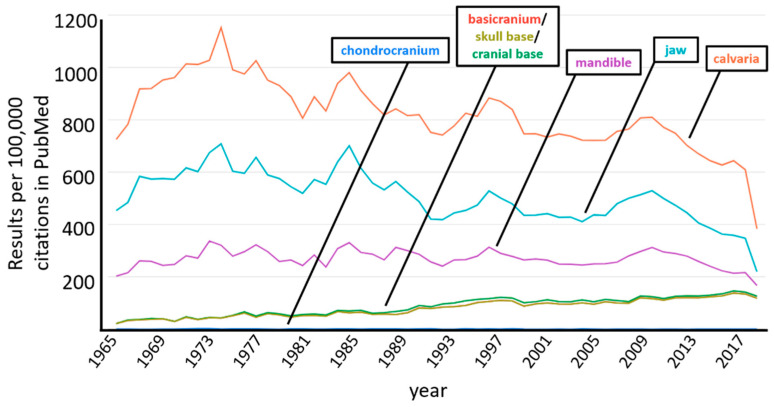
Graph depicting a keyword search of the PubMed database showing the number of articles (results per 100,000) containing the searched term (results from 1965 to 2019 shown). Note, ‘basicranium’, ‘skull base’ and ‘cranial base’ all track together, while ‘chondrocranium’ is indistinguishable from baseline of the X-axis. (Generated using, PubMed by Year: http://esperr.github.io/pubmed-by-year).

The cranial base, although inconspicuously tucked within the core of the cranium, functions almost as an anatomical girder. The cranial base supports the brain and the overlying cranial vault, while the viscerocranium (facial skeleton) largely ‘hangs’ from its rostral end. Owing to its proximity and co-development with cranial sense organs—the otic, optic, and nasal placodes—the cranial base develops in an almost ‘jigsaw-like fashion’, accommodating, protecting, and supporting these specialized organs. Likewise, given its anatomical location within the centrum of the head, just inferior to the brain, the cranial base is traversed by numerous nerves and blood vessels through its many foramina. Like the cranial vault above, the completed cranial base is a near seamless integration of tissues derived from mesodermal or ectomesenchymal neural crest cell origin. However, in stark contrast to the calvaria or the neural crest cell derived viscerocranium—which undergo a process of intramembranous ossification—the cranial base implements a ‘long bone strategy’ involving endochondral ossification. The embryonic development of the cranial base begins as a variety of separate cartilages, which fuse to form the singular chondrocranium that eventually undergoes endochondral ossification. Our understanding of the cranial base, relative to the easily accessible viscerocranial or neurocranial calvarial elements, is lacking (see Figure 1), especially at a high-level molecular resolution; this is partly due to the cranial base’s central placement within the skull. Nonetheless, because of the anatomical proximity of the cranial base with viscero- and neurocranial elements, developmental anomalies impacting the cranial base often manifest viscero- and neurocranial defects. As such, expanding our knowledge of this concealed structural element will provide broad insight into the development, disruption, and evolution of the craniofacial complex as a whole [1].

**Figure 2:**
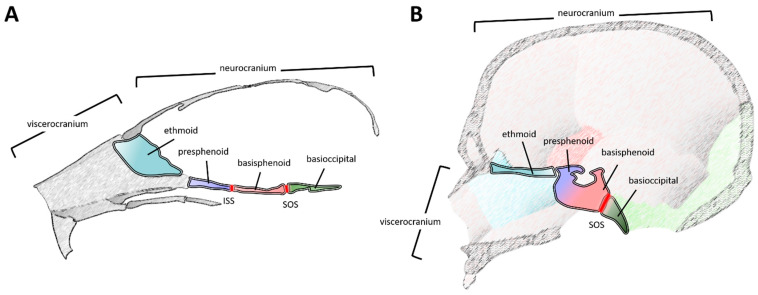
Schematics illustrating a midsagittal plane of the cranial base, in relation to other cranial structures, in a mouse (**A**) and a human (**B**). Note, scales are not equivalent. The major elements within this plane are the ethmoid (teal), presphenoid (light blue), basisphenoid (light red), and basioccipital (green). Major growth zones (synchondroses) are highlighted (dark red), including the inter-sphenoid and spheno-occipital synchondrosis. Note, in panel B, the presphenoid and basisphenoid have fused into the sphenoid (gradient of light blue and red), so the inter-sphenoid synchondrosis is not demarcated. The relative units of the skull, including the neurocranium (calvaria and the cranial base) and the viscerocranium (facial bones) are denoted. Rostral is to the left and caudal is to the right. The lower jaw (mandible) is not depicted. Abbreviations: ISS, inter-sphenoid synchondrosis; SOS, spheno-occipital synchondrosis.

Here, we briefly review the developmental origins and progression of the mouse (Part 2.1, Figure 2A) and human (Part 2.2, Figure 2B) cranial base, highlight recent loss-of-function mouse models with cranial base defects and the human pathology associated with variants in the orthologous gene (Part 2.3), and finally, conclude with a perspective on the future of cranial base research (Part 3). Rather than all encompassing, this review is meant to extend and complement previous reviews of the cranial base [2,3,4,5,6].

## 2. Development and Developmental Origins of the Cranial Base—The Mouse and Chick as a Template

**Figure 3:**
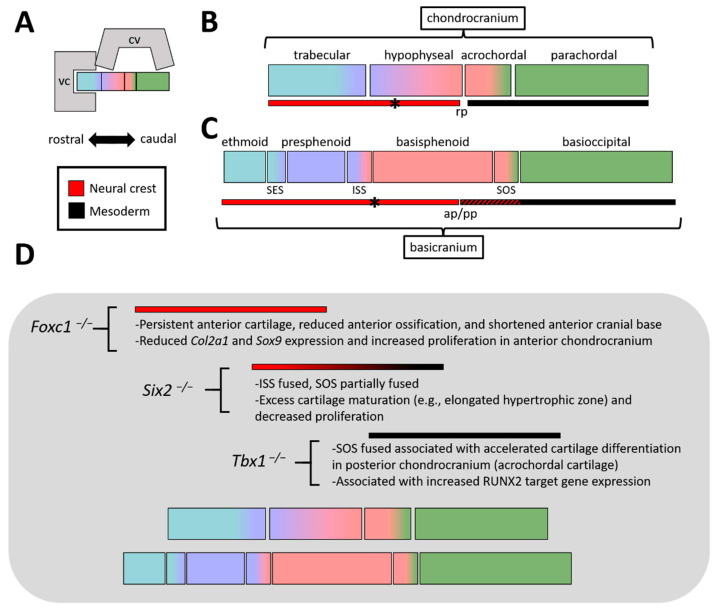
(**A**) (**Top**) Block schematic of viscerocranium, calvaria, and chondro/basicranium. (**Bottom**) Key for panels B, C, and D, highlighting orientation of rostral-caudal axis and neural crest—mesoderm color scheme. (**B**) Basic diagram of chondrocranial elements (along a midsagittal plane), highlighting the crisp neural crest—mesoderm interface at Rathke’s pouch (rp). The exception to this boundary is the hypochiasmatic cartilages (denoted by asterisk), which are mesoderm derived. (**C**) Same as B, but highlighting elements found within the basicranium (similar to Figure 2, just schematized). Note, the shades of blue are meant to provide a relative reference to their chondrocranial precursors, although boundaries are not meant to be exact. Also highlighted are the synchondroses (e.g., SES, ISS, SOS). The neural crest—mesoderm interface is found near the anterior-posterior pituitary (ap/pp), although this interface is substantially intermixed (indicated by red/black hash) within the basicranium. (**D**) Summary of the phenotypes in the 3 loss-of-function models covered in this review and where defects are located relative to the cranial base. Abbreviations: ap, anterior pituitary; cv, calvaria; ISS, inter-sphenoid synchondrosis; pp, posterior pituitary; rp, Rathke’s pouch; SOS, spheno-occipital synchondrosis; vc, viscerocranium.

Although described in exquisite detail elsewhere [7], briefly, the cartilage anlage of the cranial base (a subset of the chondrocranium) is composed of 14 paired cartilage elements—developing near the viscerocranium and calvaria (Figure 3A). These cartilage elements follow a genetically determined and spatio-temporally orchestrated developmental program, including their staggered appearance from undifferentiated mesenchyme into identifiable, condensed, chondrogenic structures. First, the caudal elements appear (e.g., parachordal cartilages). Second, the rostral elements become evident (e.g., trabecular cartilage in the nasal capsule). Finally, the midline chondrogenesis (e.g., hypophyseal and caudal trabecular cartilages) culminates in a continuous rostral-to-caudal ‘cartilaginous platform’ (Figure 3B). In addition, cartilaginous ‘struts’ (e.g., ala temporalis cartilage) and lateral elements (e.g., parietal and frontal cartilages) develop from or adjacent to this core structure, ultimately integrating with the inferior lateral components of the cranial vault. A subset of these elements also encase, protect, and support the cranial sense organs—including nasal, occular, and auditory apparatus—another key function of the cranial base. In the mouse, the process of chondrocranial development begins near embryonic day (E) 11.0 and by E16.0 the individual cartilage elements have fused into a singular structure. Again, the formation of this intermediate cartilage template is unique among the other intramembranous elements of the craniofacial skeleton, which do not utilize a cartilage intermediate.

It is from this cartilage anlage that the bony elements of the cranial base emerge through endochondral ossification. Often, a collection of fused cartilaginous elements contributes to the final bone. From rostral to caudal, the core of the cranial base is composed of the ethmoid, the sphenoid (pre- and basi-sphenoid), and the basioccipital (occipital) bones (Figure 2A and Figure 3C). Components of the frontal and temporal bones also contribute to anterior and lateral aspects of this structure, respectively. Several bones of the cranial base are separated by a synchondrosis (i.e., primary cartilaginous joint)—contributing to sagittal growth of the cranial base [6] (Figure 2A and Figure 3C). Like long bone growth plates, the cranial base synchondroses contain resting, proliferative, and hypertrophic chondrocyte zones. However, the distinction is that these zones are situated like a mirrored image growth plate with a central resting zone and bidirectional proliferative and hypertrophic zones. In the rostro-caudal direction, two major bidirectional synchondroses within the cranial base are the intersphenoidal synchondrosis (ISS) and the spheno-occipital synchondrosis (SOS), located between the presphenoid:basisphenoid or basisphenoid:basioccipital boundaries, respectively (Figure 2A and Figure 3C). In addition, a rostrally located, spheno-ethmoidal synchondrosis (located between the ethmoid and presphenoid) and an ethmoseptal synchondrosis (located between the ethmoid and nasal septum) have been described [7,8,9]. These rostral synchondroses grow in a unidirectional or radial, rather than a mirrored bidirectional, manner, likely influencing final placement of the viscerocranium [9].

While various cranial base bones and synchondroses continue to grow peri- or postnatally, they eventually cease growth in an ordered manner (e.g., ISS then SOS) at defined timepoints. Investigating postnatal trajectories of cranial base bones and synchondroses in inbred strains of mice provide a temporal description of these processes [10,11]. While the cranial base increases marginally in width, most growth is longitudinal—along the anterior-posterior (i.e., rostral–caudal) axis. Elements rostral of the presphenoid (e.g., ethmoid) complete their growth by ~postnatal day (P)7–P14 [4,11]. In contrast, regions including, and caudal to, the presphenoid continue growth up to ~P60–P120 [4,11] with cessation of individual bone growth following a sequential anterior to posterior pattern [4]. The synchondroses also exhibit postnatal growth and proliferation, although as the mice age, these active synchondroses begin to fill in with bony projections, eventually forming a continuous bridge between their rostral to caudal limits. Studies in male mice revealed the ISS fuses (i.e., formation of a continuous bony bridge) by P56 and the SOS by ~P84–P90 [4]. These timeframes are pushed later in a separate study examining female mice on a different genetic background, suggesting sex and/or strain dependencies in temporal execution of these events [11]. Finally, cranial base angle, or flexion, decreases from P7–P21 [4,11]—also known as retroflexion, this process is distinct from primates which show an increase in cranial base angle postnatally.

The germ- and tissue layer contribution of cells to the cranial base during embryogenesis has largely been defined by two animal model systems—the chick and the mouse (for an in-depth review, see [12]). Initial insight within the chondrocranium emerged from classical studies using chick-quail chimeras—allowing lineage labeling by tissue transplants and histological identification. These early studies revealed an anterior neural crest cell component, a posterior mesoderm component (of cranial paraxial and somitic origin), with an interface mapped at the centrally located basisphenoid (i.e., the basi-presphenoid and basi-postsphenoid in the chick) [13,14,15,16,17]. Specifically, the neural crest–mesoderm boundary was identified within the rostral-caudal midline of the Sella turcica; a structural feature of the basisphenoid that houses the pituitary gland and coincides with the rostral tip of the notochord [16].

These relationships were largely confirmed, and in some cases extended, in the mouse using a genetic lineage labeling approach, in which cells of neural crest or mesoderm origin were indelibly labeled with ß-galactosidase [7]. At early embryonic stages (E10.5), a neural crest (rostral)-mesoderm (caudal) interface was observed at the level of Rathke’s pouch (the future anterior pituitary gland) (Figure 3B,C). As development of the chondrocranium proceeds (~E14.5–E15.5), this interface remains at the hypophyseal-acrochordal cartilage boundary—a region ventral to the developing pituitary gland. Although sharply demarcated at the level of the chondrocranium, as ossification of the basisphenoid ensues (e.g., E17.5), these once crisp borders are softened. For example, the basisphenoid now enlists a mix of both neural crest cell and mesoderm derived osteoblasts to execute its mineralization [7]. Likewise, the neural crest-mesoderm interface within the periosteum—that is, the tissue surrounding the developing basisphenoid—shifts either rostral, on the dorsal surface, or caudal, on its ventral surface. Thus, a straight neural crest-mesoderm boundary, perpendicular to the rostral-caudal axis of the cranial base, no longer exists. While rostral synchondroses are of neural crest origin, studies in the mouse revealed that the SOS is a neural crest-mesoderm mix—neural crest found within its rostral limits while mesoderm traversed its entire caudal to rostral boundaries [7]. Finally, studies in the mouse revealed an ‘exception to the rule’ for the notochordal landmark of the neural crest-mesoderm boundary. The hypochiasmatic cartilages, located rostral to the notochord and caudal to the orbits, are of mesoderm origin (potentially the prechordal plate) [7]. This cartilage ultimately becomes integrated into the sphenoid bone and surrounded by neural crest derived elements. Thus, while most of the neural crest derived cranial base is rostral to the notochord—as predicted by the ‘New Head Hypothesis’ [18]—this exception exists.

In sum, chick, mice, along with additional organisms have provided a wealth of information concerning the development and embryonic origins of the cranial base. Like other structures within the cranium, development of the cranial base requires navigating a genetic, cellular, and morphological integration of neural crest and mesoderm populations into a seamless, functional whole. However, in contrast to these other cranial structures, the cranial base executes this process within the context of a cartilage anlage and endochondral ossification. Whether this entails novelties in the gene regulatory networks (GRN) driving these processes in the cranial base or they are replicates of previously established GRNs remains to be seen.

## 3. Pre- and Post-Natal Development of the Human Cranial Base

In contrast to the experimental accessibility of model organisms, our understanding of the ontogeny of the human cranial base (Figure 2B) is derived from cephalometric analyses of lateral head radiographs of growing individuals; investigation of human fetuses and dry skulls; and recently with the use of three-dimensional computed tomographic (CT) and magnetic resonance image (MRI) scans. These studies have investigated the pre- and post-natal growth of the cranial base by measuring the changes in sagittal (length), transverse (width), and vertical (height) dimensions as well as cranial base flexion at defined time points. These dimensional changes of the cranial base have allowed us to gain deeper insights into the ontogeny of the cranial base in pre- and post-natal development. In humans, the formation of the chondrocranium from separate cartilage elements occurs during the 6th to 8th week of gestation [19,20,21]. The cranial base length reaches 56% of the adult size pre-natally (by 38 weeks of gestation), 70% of adult size by two years of age, and the full adult size prior to adolescence [22]. Pre-natally, the human cranial base shows a differential growth pattern, and this has been quantified using high-resolution MRI of human fetuses between 11 to 23 weeks of gestation. During this time, the rate of growth of the anterior cranial base is twice that of the posterior cranial base. Additionally, the growth rate of the posterior cranial base width exceeded those in length [23]. Between the second and third trimesters of gestation, in vivo investigation of MRI scans of human fetuses has also revealed a significant increase in the sagittal and vertical dimension of the cranial base [24]. Interestingly, between the second and third trimesters of gestation, the area of the naso- and oro-pharyngeal spaces also demonstrated significant growth, which was correlated to the development of the anterior cranial base [24]. The development of these spaces is essential for speech and swallowing. Hence, growth of the cranial base—particularly the anterior cranial base—may have an influence in the development of these spaces, in addition to its role in providing structural support. From an evolutionary standpoint, the prevailing notion is that, in humans the flexion of the cranial base allows for larger brain size, relative to cranial base length [25]. This flexion could be quantified by measuring the angle between the anterior and posterior cranial base—commonly referred to as cranial base angle. The cephalometric analyses of lateral head radiographs of human fetuses between nine and 40 weeks of gestation has revealed that the cranial base angle demonstrated a statistically significant increase from the second to third trimester period. Additionally, during this period, the cranial base length (anterior and posterior) was positively correlated with maxillary protrusion and the cranial base angle was negatively correlated with mandibular protrusion, suggesting a possible influence of the cranial base on the facial skeleton [26].

Like pre-natal development, a differential growth pattern does exist during post-natal development of the cranial base. Buschang and co-workers investigated 663 lateral cephalograms from a mixed longitudinal sample of males and females between four and 16 years of age. The study results showed—albeit, individual variation existed in differential postnatal growth patterns—there was a gradient of maturity in the ontogeny of the cranial base with the anterior cranial base maturing (i.e., ceasing growth) earlier than the posterior cranial base [27]. The sequential fusion of cranial base synchondroses, probably, contributes to this maturity gradient in the cranial base: ISS fuses between two and three years; spheno-ethmoidal synchondrosis fuses between 9 and 15 years; SOS fuses between 16 and 18 years of life [3]. These patterns largely mimic those seen in the mouse, where 2–3 (human) years is equivalent to ~P28 (early stages of ISS fusion in the mouse) and 16–18 (human) years is equivalent to ~P60–70 (general timeframe of SOS fusion in the mouse, also correlating with stages of puberty, as in humans) [4,11,28].

Furthermore, it is important to consider the onset of adolescent growth, peak growth velocity, and cessation of adolescent growth of the cranial base in relation to other craniofacial bones—particularly the viscerocranium (maxilla and mandible). In this regard, the evaluation of Fels Longitudinal Study samples—comprised of 293 untreated boys and girls at the age of 4–24 years—has provided interesting insights into the onset, peak velocity, and cessation of growth for cranial base length, cranial base angle, maxillary length, mandibular length and gonial angle. This study identified that, in comparison to cranial base length, growth of the maxilla and mandible showed an earlier age of onset but a later age of peak velocity and growth cessation [29]. Rephrased, Nahhas et al.’s study indicates that the onset of cranial base growth is after that of the maxilla and mandible, however, the peak velocity and cessation of cranial base growth is achieved earlier than that of the maxilla and mandible. Therefore, there is a temporal window during post-natal development, in which cranial base growth potentially influences viscerocranial growth. In addition, Nahhas et al. also identified that cranial base angle decreased with age during adolescence [29].

Both human and animal studies have provided evidence suggesting that growth of the cranial base can influence growth of the facial skeleton—a subset of which we highlight here. Firstly, the immobilization of cranial base sutures by methyl cyanoacrylate in New Zealand White Rabbits has shown that craniofacial length was significantly shortened when the cranial base is fused [30]. Secondly, the cephalometric study of 11 anencephalic fetuses compared with 60 normal fetuses discovered a significant reduction in cranial base length and an acute cranial base angle associated with a reduction in the relative size of the cranium and a flattening of the calvarium. Further, the forward displacement of the nasomaxillary segment observed in normal fetuses did not occur in anencephalic fetuses, potentially a consequence of disrupted cranial base (and/or calvarial) development [31]. This is clinically important as these cranial base growth anomalies—and their impact on the growth of the nasomaxillary complex—could manifest as a retrusive position of the maxilla and an underbite. This phenotype is significant, as patients with a retrusive maxilla often require an orthopedic or surgical correction to position the maxilla in a proper forward position. Ultimately, this procedure would improve masticatory function and possibly improve airway dimensions. Thirdly, studies that have investigated crania that are artificially deformed by applying compressive forces—a cultural practice common among certain ethnic groups across the globe—provided insights into the modularity and integration between anatomical units, particularly the neurocranium and viscerocranium [32]. In the artificially deformed skull, the cranial base showed a discernable abnormality, and this produced a restricted antero-posterior growth of the naso-maxillary complex [33]. Finally, investigation of morphological covariation between the face and the cranial base (midline and lateral) in groups of children (6–10 years) and adults (20–35 years) has further demonstrated morphological integration between these elements. In children, moderate correlation between the midline cranial base and face has been discovered. However, this correlation was reduced in adults. These data indicate that the integration between craniofacial structures exists and this could be influenced by the duration of common developmental timing [34]. Collectively, these studies suggest that the cranial base in humans—in addition to providing structural support for the developing brain—plays a pivotal role in the sagittal positioning of the nasomaxillary complex. The integration of these structures is an important consideration in the context of human conditions exhibiting cranial base anomalies.

## 4. Genetic Control of Cranial Base Development

**Figure 4:**
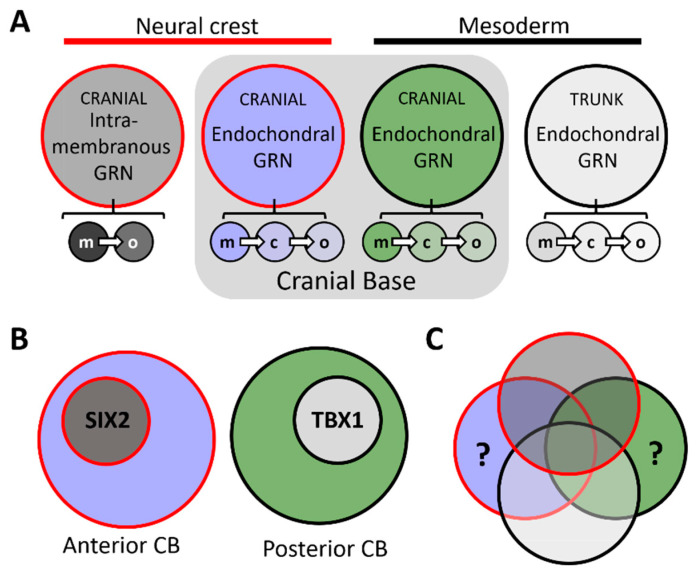
(**A**) Diagram representing hypothetical gene regulatory networks (GRNs) for neural crest intramembranous (red outline, dark grey fill), neural crest endochondral (red outline, light blue fill), cranial mesoderm endochondral (black outline, green fill), and trunk mesoderm endochondral (black outline, light grey fill) modes of ossification. Note, while each large circle represents a collective of GRNs, these processes could be broken down into cell-type specific GRNs, including ‘mesenchymal precursor’ (m), chondroblast/cyte (c), and osteoblast/cyte (o)—represented by the small circles in A. (**B**) The prevailing notion would suggest that anterior or posterior cranial base GRNs are composed of reutilized GRN ‘modules’ from other tissues. For example, the anterior cranial base ‘SIX2 module’ [52] is the same that is used in neural crest derived viscerocranial elements. Likewise, for the posterior cranial base ‘TBX1 module’ [68] and other TBX1 GRNs. (**C**) Venn diagrams conceptualizing GRN overlap between these different skeletal populations. Whether novelties exist for anterior and posterior cranial base GRNs (regions denoted by ‘?’) remains to be fully realized. Abbreviations: c, chondroblast/cyte; CB, cranial base; GRN, gene regulatory network; m, mesenchymal precursor; o, osteoblast/cyte.

Relative to the viscerocranium or cranial vault, the molecular control of cranial base development—including anterior, posterior, or as a whole—is not well defined. However, the chick and murine models have provided distinctive insights into the ontogeny of the cranial base. Given the embryological origins (neural crest/mesoderm, described in Section 2 above) and developmental processes (i.e., endochondral ossification) of the cranial base, it is not surprising that genetic mutations disrupting its formation often coincide with additional congenital anomalies. For example, disruptive gene mutations impacting the process of endochondral ossification can affect not only the cranial base but also other endochondrally formed bones of the axial and appendicular skeleton. Likewise, while the anterior cranial base may be impacted by mutations disrupting neural crest cell development, these mutations will often coincide with additional cranial neural crest associated anomalies of the viscer- or neuro-cranium. Previous reviews have adequately summarized a number of these genes and pathways, often focusing on signaling molecules (e.g., FGFR, IHH, BMP, etc.) regulating cranial base development [2,3,4]. Here, we briefly highlight a subset of loss-of-function models of transcription factors that preferentially impact the anterior or posterior cranial base in the mouse—a system that shares several developmental trajectories of human chondrocranial and craniofacial development [35]. While these examples provide evidence of transcription factors integrated into a cranial base GRN, an in depth, unbiased, molecular characterization will provide a more comprehensive picture of this GRN (e.g., the direct transcriptional targets of these factors in the cranial base). Comparing cranial base GRNs to both trunk endochondral and cranial neural crest cell intramembranous GRNs will help in defining what novelties, if any, exist for these networks specifically in the basicranium [36] (Figure 4).

### 4.1. Foxc1, Six2, and Tbx1—Anterior to Posterior Control of Cranial Base Development

#### 4.1.1. FOXC1

FOXC1 is a member of the forkhead, boxed, winged, helix protein family—a family of highly conserved transcription factors. These proteins regulate developmental processes of cell fate specification, proliferation, and differentiation [37]. Examination of control and *Foxc1* spontaneous mutant (hydrocephalous [38]) cranial skeletons just prior to birth (E18.0) revealed a preferential impact on the ossification of the presphenoid (i.e., persistent caudal trabecular cartilage) and midline basisphenoid in mutants, relative to controls (Figure 3D). In stark contrast, except for slightly expanded SOS, the components of the posterior cranial base in mutants (e.g., basioccipital) were largely intact and unaffected as in controls. Chondrocranial defects in mutants were already evident in similar anatomical locations at earlier developmental timepoints (e.g., E15.5). For example, the caudal trabecular cartilage was already foreshortened and the midline of the hypophyseal cartilage was absent in mutants, relative to controls. Consistent with a defect in these structures, analysis of *Foxc1* transcripts revealed a robust expression of its mRNA within the anterior mesenchymal condensates, subsequent chondrocranial elements, and the perichondrium of forming bones. Further, molecular analysis of the cranial base revealed that loss of *Foxc1* was associated with reduced expression of chondrogenic markers (*Sox9* and *Col2a1*) concomitant with a significant increase in cell proliferation—defects that were observed in the anterior, but not posterior, hypophyseal cartilage of mutants relative to controls. These findings highlight the specificity of FOXC1 in anterior cranial base development.

In line with observations in the mouse, loss of *FOXC1* in humans is associated with Axenfeld-Rieger Syndrome (ARS) and pathogenic variants in this gene have been identified in ARS patients (MIM 180500) [39,40,41]. ARS is a rare autosomal dominant syndrome (1;200,000) with a phenotypic presentation involving ocular, dental, and umbilical defects as well as craniofacial dysmorphisms [42,43,44,45,46]. The discernable craniofacial presentation of these patients includes maxillary hypoplasia with a concave profile, which is commonly referred to as a Class III skeletal presentation [42,43,44,45,46]. The cephalometric measurements on lateral head radiographs of ARS patients, compared to normative standards, has shown changes in cranial base length and/or cranial base angle [42,43]. In addition, abnormal Sella turcica morphology is also seen in ARS patients [47]. The maxillary hypoplasia phenotype seen in ARS patients has been largely believed to be due to missing teeth or defects in neural crest cells. However, as described earlier, the influence of cranial base growth on the naso-maxillary complex suggests that maxillary hypoplasia seen in ARS patients could be partly due to cranial base abnormalities (manifested as changes in cranial base length and/or angle). The results of murine studies [38] highlighted above provide additional support for the role of cranial base changes contributing to maxillary hypoplasia phenotypes observed in ARS patients.

Finally, it is interesting to note two aspects of FOXC1 during development. First, although defects are isolated to the anterior (i.e., neural crest derived) cranial base in *Foxc1* mouse mutants, *Foxc1* is initially expressed (~E7.5) at high levels in both the presomitic and head mesoderm, along with the cranial neural crest [48]. Second, it has been observed that trunk skeletal endochondral ossification is regulated by FOXC1. In this regard, FOXC1 was shown to be expressed in long bone chondrocytes and its loss was associated with delayed ossification of limb bones [49]. Further, FOXC1 was shown to physically interact with GLI2, downstream of IHH signaling, to activate expression of IHH responsive genes (e.g., *PTHrP* and *Col10a1*)—necessary for proper endochondral ossification [49]. In lieu of these findings it is interesting to note that loss of IHH signaling preferentially impacts the anterior cranial base, in part due to compensating SHH signaling in the posterior cranial base [50]. However, even if FOXC1 coordinates with GLI-family members downstream of IHH/SHH signaling in the cranial base, different dependencies must exist given the preferential anterior defects observed in *Foxc1* mutants. One possibility is that compensating posterior factors (e.g., different FOX paralogs) exist. How signaling pathways converge on FOXC1 within different axes of the cranial base during development remains to be seen.

#### 4.1.2. SIX2

*Six2* is a member of the sine oculis/Six family of genes encoding numerous transcription factor paralogs. The SIX family of proteins regulate cell proliferation and survival during embryogenesis [51]. Inactivation of *Six2* in mice was identified to cause prominent anterior with subtle posterior cranial base defects, as determined by bone and cartilage staining [52] (Figure 3D). Notable in *Six2* mutants, relative to controls, was the entire fusion of the presphenoid to the basisphenoid in newborn pups, associated with a complete ablation of the ISS. Although not as severely impacted, the basisphenoid and basioccipital, also showed partial fusion due to the mineralization of the SOS in mutants relative to controls. Highlighting the specificity of these defects, the mandible, calvaria, occipital and parietal regions were largely normal, although the upper face was retracted in mutants relative to controls—potentially due to the foreshortened cranial base. Again, earlier time points of chondrocranial development were investigated (e.g., E14.5), revealing excessive maturation of cartilage (e.g., extended zone of hypertrophic chondrocytes) coupled with reduced proliferation within the mutant versus control anterior cranial base (e.g., cartilage condensate of the presphenoid). Further, ossification per se did not appear affected in mutants, relative to controls. However, the proliferation defects coupled with extended differentiated chondrocytes in mutants likely exhausted the synchondrosis and provided additional template for ossification. Like *Foxc1* expression patterns, investigation of *Six2* transcripts revealed prominent mRNA expression within the anterior cranial base (presphenoid and anterior basisphenoid). This finding was supported by analyzing *SIX2* expression in human embryos, revealing a likely autonomous role for SIX2 within this structure. This notion was recently supported by targeted deletion of *Six2* within the anterior cranial base, which largely recapitulated the cranial base defects found in the complete *Six2* knockout [53].

It was appreciated during initial observations of the *Six2* mouse mutant phenotype that the midface defects were reminiscent of the human condition, Frontonasal dysplasia (FND). FND is a congenital disorder, which encompasses a spectrum of craniofacial phenotypes with three distinct subtypes: FND1, FND2, and FND3 (MIM 136,760, 613,451, and 613,456, respectively). The FND1 patients present with an anterior cranial vault defect (anterior cranium bifidum occultum); widely separated eyes (hypertelorism); nasal clefting; V-shaped frontal hairline; slit nostrils; and cleft palate [54]. The FND2 and FND3 patients may have an additional clinical presentation of alopecia and absent or small eyes, respectively [54]. A new type of FND was reported with a clinical manifestation of frontal bossing, high hairline, ptosis, hypertelorism, broad nasal tip, large anterior fontanelle, sagittal synostosis, conductive hearing loss, and cranial base anomalies [55,56]. These FND patients carried deletions in the genomic region that contains the *SIX2* gene [55,56]. The cranial base anomalies observed in this new type of FND included persistent craniopharyngeal canal and premature lateral SOS [55]. To what degree are the cranial base anomalies causing and/or influencing the additional defects observed in these FND patients or are they isolated pathologies? Additional studies will be required to help decouple the relatedness of these defects.

Finally, although loss of *Six2* does not appear to impact trunk skeletal development (as did loss of *Foxc1*), *Six2* is expressed during early cranial neural crest development [57] and several neural crest derived structures are SIX2 dependent. For example, proper development of the neural crest derived middle ear ossicles [56,58] and palatal shelves [59], require adequate SIX2 levels. Thus, specificity of the anterior cranial base defects in *Six2* mouse mutants is likely due to SIX2′s role in a broader neural crest GRN (Figure 4B)—although direct versus indirect influences are difficult to decouple. Along with the neural crest, *Six2* is expressed in pharyngeal [60] and trunk mesoderm (e.g., kidney) [61,62,63] populations, where it also functions. Interestingly, trunk *Six2* expression is regulated by both WNT and BMP signaling [64], two signaling systems necessary for cranial base development [65,66]. It will be interesting to identify if SIX2 functions in the neural crest after an intramembranous versus endochondral fate decision has been made, and if so, whether the direct transcriptional targets of SIX2 have diverged (i.e., the degree of the GRNs not overlapping) between neural crest fated for both modes of ossification (Figure 4B,C). Whether regulators of *Six2* expression in the trunk mesoderm (e.g., WNT, BMP) are utilized in the cranial neural crest will also require further investigation.

#### 4.1.3. TBX1

TBX1 is a member of the T-box transcription factor family—a protein family critical for differentiation, proliferation, among other processes during embryonic development [67]. The loss of *Tbx1* in mice resulted in posterior cranial base defects, including the ectopic mineralization of the SOS [68] (Figure 3D). Premature mineralization resulted in the fusion of the basisphenoid and the basioccipital bone. Congruent with the mesoderm contribution to the SOS, conditional deletion of *Tbx1* within the mesoderm (Mesp1-CRE) recapitulated the SOS defects observed upon global deletion of *Tbx1*. In contrast, neural crest cell specific (Wnt1-CRE) loss of *Tbx1* did not impact the anterior cranial base, highlighting the spatial specificity of TBX1 function in the cranial base. Consistent with the posterior cranial base defects, expression of *Tbx1*—in this case using a *Tbx1*-*CRE* lineage tracing approach—revealed extensive labeling (i.e., *Tbx1* expression) in the posterior, mesoderm derived, chondrocranium (e.g., acrochordal and parachordal cartilages). In contrast, labeling was not detected in the anterior chondrocranium, including the ISS. Closer examination of the developing posterior chondrocranium, the acrochordal cartilage, revealed abnormal chondrocyte differentiation in *Tbx1* mutants relative to controls. Further, through a combination of biochemical and in vivo studies, a molecular network was uncovered in which TBX1 interacted with and repressed RUNX2 activity [68]—a key transcription factor in orchestrating the cartilage to bone transition during endochondral ossification. Loss of *Tbx1* thus corresponded with elevated expression of RUNX2 target genes—including *Spp1*, *Col1a1*, *Vegfa*, and *Hapln1*—in the acrochordal cartilage, relative to controls. In turn, this dysregulated GRN created accelerated chondrocyte differentiation followed by loss of the SOS.

In humans, genetic studies have identified the *TBX1* gene as likely contributing to the pathogenesis of Velocardiofacial syndrome (VCFS) (MIM 192430) [69,70]. VCFS, also known as the 22q11.2 deletion syndrome (22q11.2DS), is a congenital disorder with a clinical manifestation of cardiac defects, learning disability, dysfunction of the immune system, and characteristic craniofacial dysmorphisms such as long face, cleft palate, microcephaly, retrognathia, broad nose, and ear anomalies. Consistent with the role of the *Tbx1* gene in murine posterior cranial base development [68], cephalometric and MRI studies have identified cranial base anomalies in VCFS patients. Interestingly, the 22q11.2DS patients exhibit a reduced cranial base length and an enlarged cranial base angle with a retrognathic position of the mandible in relation to the maxilla [71,72,73,74]. Again, decoupling the cause and effect of defects in different anatomical structures will require additional investigation—including the use of conditional mouse genetics.

Is TBX1 part of a unique posterior cranial base GRN (Figure 4B,C)? Expression of *Tbx1* is largely isolated to mesoderm populations [75,76]. Thus, the specificity of posterior defects in the cranial base likely reflects this expression domain. Given the defects observed in other mesoderm structures (e.g., cranial and cardiac muscles), TBX1’s role in the posterior cranial base could simply be the deployment of a broader, core mesodermal GRN (Figure 4B)—alternatively, unique posterior cranial base transcriptional targets of TBX1 are possible (Figure 4C). Like the previous transcription factors highlighted, developmental timing is a key concept in formulating hypotheses about these networks. For example, if TBX1 functions in an early mesoderm precursor, its transcriptional targets (i.e., its GRN) are likely shared between TBX1-dependent tissues. However, if TBX1 functions exist within posterior cranial base cell populations (e.g., once specified to an endochondral cartilage or bone fate), unique transcriptional targets are plausible—an exception being genes that regulate basic cellular processes, such as cell proliferation. It is interesting to note that while most studies have placed TBX1 function within the mesoderm, neural crest specific deletion of *Tbx1* resulted in defects to the neural crest, endochondral-derived, hyoid bone [77]—extending TBX1′s function in bone development beyond the mesoderm. Are transcriptional targets of TBX1 shared between the posterior cranial base and the hyoid? Are upstream regulators of *Tbx1* expression, such as SHH [78], utilized in both locations? Again, a higher resolution molecular profile will help resolve these questions.

Although limited in scope, the examples of *Foxc1*, *Six2*, and *Tbx1* loss-of-function models highlight transcription factors regulating cranial base development—including regulation with anterior or posterior specificity. Undoubtedly, these transcription factors are deployed in response to external signaling programs already implicated in cranial base ontogeny, likely facilitating their anterior to posterior expression domains and localized functions. Both human genetics and animal models have revealed the loss of genes regulating cranial base development coincide with broader anomalies—a feature stemming from the cranial base sharing regulatory networks with the neural crest and endochondral ossification (Figure 4A,B). While the prevailing notion would suggest these programs are executed in a similar fashion between the cranial base and these other tissues (e.g., the direct transcriptional targets of a transcription factor in the anterior cranial base are the same targets of the transcription factor in viscerocranial elements, or the networks regulating endochondral ossification are identical between the cranial base and elements of the trunk skeleton) (Figure 4B), the extent of novelties that exist (Figure 4C), remains to be seen.

## 5. Concluding Remarks

Several elegant studies, ranging from animal developmental biology to human anthropology, have assembled a framework of cranial base anatomy, embryology, molecular genetics, evolution, and dysfunction. However, despite this compendium of experimental work, the cranial base remains relatively understudied, especially in comparison to the cranial vault and facial skeleton—despite its close integration with these superficial elements. Continued investigation and knowledge of the cranial base will be aided by the ever-expanding experimental toolkit, including novel molecular/genomic techniques, accessible non-traditional animal models due to gene-editing approaches, and quantitative imaging methods, among others. Such techniques will provide the ability to revisit and refine long-standing models, while asking novel biological questions and uncover new facets of the cranial base. As an example, the advent of single cell genomics provides a platform to lineage trace cell populations—using de novo spontaneous mutations—in post-mortem tissues [79]. Such methods could help clarify the neural crest-mesoderm interface of the cranial base in primates, including humans, and in comparison to previous models (chick, mouse) assess if and how this boundary has shifted during evolution. As a second example, the coupling of genome wide approaches (e.g., genome-wide association studies) with detailed measurements of cranial base traits, has provided the identification of associated loci that may harbor novel genes, or non-coding elements, influencing cranial base features [80]. While only the ‘tip of the iceberg’, these emerging techniques will provide depth to our understanding of the cranial base.

Further, with a focus on cranial base embryology, it will be important to define both the signaling centers and the plasticity of responding cells within different axial limits of the cranial base. This is particularly true for the anterior cranial base. While the notochord has been shown to provide essential signals needed for posterior cranial base development [50], less is known regarding the exact signals and signaling sources critical for the initiation and progression of anterior cranial base development. In the same vein, the intrinsic (i.e., cell autonomous) GRNs that respond to these signals and elicit an endochondral ossification response are not well understood. What are the mechanisms by which a pool of initially homogeneous cranial neural crest cell progenitors can be driven down an intramembranous versus endochondral trajectory? Further, how malleable are cranial neural crest cells once these trajectories have been established? For example, is there a developmental timepoint in which a viscerocranial destined mesenchymal cell would no longer elicit an appropriate response if placed within the context of the anterior cranial base (or vice versa)? A similar question could be asked regarding progenitor cells of the mesoderm derived posterior cranial base and those found within, for example, a developing long bone of the appendicular skeleton. Further, is there a developmental timepoint in which anterior (neural crest) and posterior (mesoderm) cranial base GRNs converge into a similar (or identical) endochondral program, or do their distinct embryological origins always carry a molecular mark with them? This may be particularly pertinent at the boundary of the old and new head. While previous studies identified limited distinctions between, for example, trunk and cranial ossification programs (using classical markers) [81,82], these findings warrant reinvestigation given the recent expansion of techniques to unbiasedly dissect molecular programs. Again, the comparison of GRN’s between cells found in these distinct anatomical positions, over developmental time, will help parse out whether distinct molecular identities exist and if so, when they are established (Figure 4C).

Equally intriguing in this regard is gene co-option, an evolutionary and developmental biology (evo-devo) concept, as it relates to the cranial base. An idea heavily embedded in the evolution of the neural crest, gene co-option is the recruitment of GRNs from one tissue/cell-type into another [83]. This co-option is thought to occur in part through acquisition of novel transcription factor binding motifs, resulting in network activation in a novel spatial or temporal location. Given the ability of the cranial neural crest to reach a state of mineralization via two distinct mechanisms—intramembranous and endochondral—the evolutionary processes that imbued these cells with dual capabilities is of interest. Did the cranial neural crest co-opt a mesodermal endochondral program? Did the cranial neural crest independently co-opt an intramembranous program? Did one emerge from the other? Examination of a variety of extant organisms, including invertebrates (i.e., no neural crest) is helping resolve these questions in cell type and organismal transitions [84,85]. Of functional interest is the necessity of adopting an endochondral program within the cranial base when the majority of its skeletal neighbors are intramembranous. Does this reflect an evolutionary byproduct, diverged evolutionary trajectories, or does this imbue the cranial base with needed, and thus selected for, function (rigidity, flexion competence, growth rate, or some other feature of adaptability)? Such concepts have been long contemplated but are difficult to functionally test and validate. However, investigation of these questions will be important in providing additional insight into the molecular and anatomical evolution of this structure.

Finally, as an extension of the previous discussions—and a concept repeatedly touched on here and in numerous studies of the cranial base—there is need to discern the relationship of primary versus secondary effects between cranial base anomalies and anomalies found at different anatomical locations [86,87,88,89]. For example, the indirect influence of cranial base defects on the pathology of calvarial or viscerocranial elements (e.g., cleft secondary palate) becomes difficult to unlink if GRN’s are shared between these structures. One approach to try and circumvent this conundrum, specifically in the context of mouse genetics, is the use of ‘endochondral specific’ CRE-recombinase lines. Given the cartilage anlage is an inherent feature of endochondral ossification, CRE recombinase lines that can target this intermediate of endochondral bone should theoretically avoid gene deletion in intramembranous bones. However, care must be taken as genes thought to be cartilage specific (i.e., endochondral specific) have been shown to label osteo-chondroprogenitors in intramembranous bones as well (e.g., Col2a1-Cre) [90,91,92]. As such, interpreting defects in the viscerocranium or calvaria as ‘secondary’ to the cranial base becomes problematic. A cranial base specific CRE recombinase line would be an ideal approach, although, whether such a gene or non-coding element—driving cranial base specific expression—exists, remains to be seen. Here again, emerging technologies such as single cell RNA-sequencing, single cell chromatin accessibility assays, and ever-expanding computational resources, may help in identifying whether a cranial base-specific molecular program exists—or rather, it is a modular assembly of preexisting GRNs.

Coupled with the role of the cranial base in a variety of human congenital anomalies—including its influence on codeveloping craniofacial structures—the cranial base and its predecessor, the chondrocranium, will undoubtedly remain an active area of investigation. As with a variety of developmental systems and processes, the future is bright for research of this anatomical girder.
